# Editorial: Structural and Biochemical Aspects of the Interaction of β-Lactamases With State-of-the-Art Inhibitors

**DOI:** 10.3389/fmicb.2022.849324

**Published:** 2022-02-02

**Authors:** Krisztina M. Papp-Wallace, Jean-Denis Docquier, Frederic Kerff, Pablo Power

**Affiliations:** ^1^Research Service, Veteran Affairs Northeast Ohio Healthcare System and Departments of Medicine and Biochemistry, Case Western Reserve University, Cleveland, OH, United States; ^2^Department of Medical Biotechnologies, University of Siena, Siena, Italy; ^3^InBioS, Center for Protein Engineering, University of Liège, Liège, Belgium; ^4^Facultad de Farmacia y Bioquímica, Instituto de Investigaciones en Bacteriología y Virología Molecular (IBaViM), Universidad de Buenos Aires, Buenos Aires, Argentina; ^5^Consejo Nacional de Investigaciones Científicas y Técnicas (CONICET), Buenos Aires, Argentina

**Keywords:** Beta-lactamase inhibitors, Antimicrobial resistance (AMR), diazabicyclooctane inhibitor, boronate inhibitors, MBL inhibitors, X-ray chrystallography, molecular dynamics simulation, enzyme kinetics and inhibition

Multidrug resistance in Gram negative pathogens (i.e., Enterobacterales, *Pseudomonas aeruginosa*, and *Acinetobacter baumannii*) is a major threat to global public health. Moreover, the situation has been recognized by the World Health Organization (WHO), who stated in 2011 that “no action taken today could result in no cure tomorrow,” and from 2016 all countries were called on to promote a better use of available antibiotics and to build and execute efficient tools to prevent bacterial infections, under the “One Health” concept.

β-Lactams represent one the largest and most prescribed classes of antibiotics used to treat infections caused by Gram negative bacteria. However, over the past two decades, the efficacy of this class of antibiotics is being thwarted by the constant evolution and emergence of enzymes that can hydrolyze β-lactams known as β-lactamases (Gutkind et al., 2013). β-Lactamases are the predominant resistance mechanism present within Gram-negative bacteria and are classified into different structural (Classes A, B, C, and D) based on their overall protein fold (Ambler et al., 1991). Of these enzymes those with extended-spectrum activity (e.g., CTX-M) as well as carbapenemase activity (e.g., KPC, VIM, NDM, OXA-23, OXA-48) remain the most problematic. Thus, identifying novel inhibitors, to be used in combination with β-lactam antibiotics, as well as strategies to target these enzymes, is critical to conserve our β-lactam armamentarium (Papp-Wallace and Bonomo, 2016; Tooke et al., 2019).

Within this Research Topic, insights into the inhibition of β-lactamases by different chemical scaffolds [e.g., boronic acids, diazabicyclooctanes (DBOs)] is discussed. Moreover, the role of enzyme stability as a predictor toward the evolution of β-lactamase variants is described.

Up until 2014, our β-lactam-β-lactamase inhibitor arsenal was limited, in that, only infections caused by bacteria producing Class A [e.g., penicillinases (TEM-1, SHV-1) and extended-spectrum β-lactamases (ESBLs) (CTX-M)] were treatable with these agents. The approval of ceftolozane-tazobactam in 2014 and ceftazidime-avibactam in 2015 by the US Food and Drug Administration (FDA) expanded the clinician's toolbox as infections caused by Gram-negatives producing Class A carbapenemases (e.g., KPC) as well as Class C (e.g., AmpC, CMY) and some Class D β-lactamases (OXA-48) were now treatable with these novel β-lactam-β-lactamase inhibitor combinations (Ehmann et al., 2012; Sader et al., 2014). The release of these agents was a game-changer as well as the subsequent FDA approvals of meropenem-vaborbactam and imipenem-relebactam. Avibactam and relebactam possess a unique DBO scaffold, while vaborbactam is a boronic acid-based inhibitor; both platforms lead to potent β-lactamase inhibition. However, gaps remain in the treatment of infections caused by Gram negatives producing Class B and D carbapenemases as well as novel variants of Class A and C that are emerging in clinic since the introduction of ceftolozane-tazobactam and ceftazidime-avibactam.

Within this Research Topic, two review articles present the next-in-class DBO and boronic acid inhibitor scaffolds, durlobactam (Shapiro et al.), and QPX7728 (Lomovskaya et al.), respectively. A third review article focuses on structural studies published to date with DBO and boronic acid scaffolds using CTX-M as a model enzyme with implications for enhancing these inhibitors' profiles (Shurina and Page). Durlobactam offers additional inhibition beyond that of avibactam and relebactam as durlobactam is a potent inactivator of Class D carbapenemases and exhibits intrinsic antimicrobial activity directly inhibiting select penicillin binding proteins (PBPs). Durlobactam will be partnered with sulbactam to target infections caused by difficult-to-treat *Acinetobacter* species as sulbactam can also inhibit PBPs in this genus. Currently, sulbactam-durlobactam is being evaluated in a global Phase 3 trial (called ATTACk, for **A**cinetobacter **T**reatment **T**rial **A**gainst **C**olistin), to determine its efficacy and safety in patients with infections caused by *Acinetobacter* species; if succeeded, and it gains regulatory approval, sulbactam–durlobactam will be an important treatment option for patients with serious and life-threatening infections caused by *Acinetobacter* species, including carbapenem-resistant strains.

QPX7728 also possesses an enhanced inhibition profile as it can inhibit most Class A, B, C, and D β-lactamases. Its ultra-broad-spectrum β-lactamase inhibitory effect relies on a strong inhibition of metallo-β-lactamases while also keeping a potent serine-β-lactamases inhibitory effect. This feature makes QPX7728 suitable for being used as a stand-alone β-lactamase inhibitor, where it can be combined with multiple different β-lactams, with the goal of providing more optionality for both treatment and stewardship efforts. QPX7728's partner is still under wraps; however, it is being pursued for both intravenous and oral administration. Both combinations are currently in various stages of clinical development and are eagerly awaited additions to antibiotic formularies.

Shurina and Page's review spotlights the key structural features of CTX-M enzymes for substrate and inhibitor binding (i.e., the Ω-loop, the 240-loop, the Y105-loop, and the SDN-loop) as well as paucity of structural studies of DBOs and boronates within the CTX-M class, of which at the time of publication included 115 enzyme variants.

Two original research articles delve into the inhibition of β-lactamases by β-lactamase inhibitors with a boronic acid scaffold. This includes S02030's activity vs. MOX-1 (Ishikawa et al.), and molecular dynamics simulations of novel boronates (i.e., vaborbactam, taniborbactam, and QPX7728) with Class B metallo-β-lactamases and Class D carbapenemases (Lence and Gonzalez-Bello). MOX-1 is a Class C β-lactamase with extended-spectrum activity toward cephalosporins. Ishikawa et al. explore the structural inhibition of MOX-1 by the boronate, S02030 in comparison to aztreonam and further demonstrate potency of S02030 kinetically as well as against bacteria when S02030 is combined with ceftazidime. Using aztreonam as a comparator, the authors were able to uncover that S02030 bound deeper into the active site pocket. Conversely, aztreonam remained the more potent inactivator due to its SO_3_ group that is lacking in S02030 but allows aztreonam to form additional interactions thus improving its IC_50_ and *K*_*i*_ values against MOX-1. The authors postulate that the addition of an S0_3_ group to S02030 may improve its potency. The molecular dynamics study of OXA-48, OXA-23, and OXA-24/40 by Lence and Gonzalez-Bello. reveals that the horizontal arrangement of QPX7728 as compared to taniborbactam and other bicyclic boronates allows for superior binding to these β-lactamases. Moreover, evaluating the IMP-1 metallo-β-lactamase, they found that IMP-1 has the least flexible L10 loop compared to VIM-2 and NDM-1; this feature is likely why bicyclic boronates do not inhibit IMP enzymes as well as other metallo-β-lactamases.

An additional two original research articles round out this topic with a focus on β-lactamase stability, one examining non-active site motifs within Class A β-lactamases, hydrophobic nodes (Olehnovics et al.), and the other dissecting the mechanistic basis for the lack of inhibitor-resistant variants of the BlaC β-lactamase from *Mycobacterium tuberculosis* (Bhattacharya et al.). Olehnovics et al. conducted adaptively sampled molecular dynamics simulations to evaluate the hydrophobic nodes that stabilize the β-lactamase core using SME-1, KPC-2, SHV-1, and TEM-1 as model enzymes. They found that the overall dynamics of these class A β-lactamases are similar, which provides an explanation for why mutations far from the active site alter enzymatic activity. In the other study, using circular dichroism spectroscopy, differential scanning calorimetry, molecular dynamics simulation, and stability-based enzyme activity assays, Bhattacharya et al. determined that S130A and S130G variants of BlaC are unlikely to emerge in the clinic as these variants are much less stable, thus the beta-lactamase inhibitors, clavulanic acid, sulbactam, and tazobactam should remain active against this enzyme.

Overall, this special topic on the interactions of β-lactamases with β-lactamase inhibitors should provide the readers with some hope on the horizon as there are novel agents in the pipeline. Moreover, articles within this topic revealed mechanistic bases to design innovative inhibitors as well as aided in the understanding of the development of inhibitor resistance in β-lactamases. Bringing all these aspects together will allow for the further advancement of this field and the discovery of the next generation β-lactamase inhibitors.

## Author Contributions

KP-W, J-DD, FK, and PP contributed equally in the original conception of the special issue. KP-W and PP wrote the first draft of the manuscript. All authors contributed to manuscript revision, read, and approved the submitted version.

## Conflict of Interest

The authors declare that the research was conducted in the absence of any commercial or financial relationships that could be construed as a potential conflict of interest.

## Publisher's Note

All claims expressed in this article are solely those of the authors and do not necessarily represent those of their affiliated organizations, or those of the publisher, the editors and the reviewers. Any product that may be evaluated in this article, or claim that may be made by its manufacturer, is not guaranteed or endorsed by the publisher.

## References

[B1] Ambler R. P. Coulson A. F. Frere J. M. Ghuysen J. M. Joris B. Forsman M.. (1991). A standard numbering scheme for the class A beta-lactamases. Biochem. J. 276, 269–270. 10.1042/bj2760269 2039479 PMC1151176

[B2] Ehmann D. E. Jahic H. Ross P. L. Gu R. F. Hu J. Kern G.. (2012). Avibactam is a covalent, reversible, non-beta-lactam beta-lactamase inhibitor. Proc. Natl. Acad. Sci. U.S.A. 109, 11663–11668. 10.1073/pnas.1205073109 22753474 PMC3406822

[B3] Gutkind G. O. Di Conza J. Power P. Radice M. (2013). β-Lactamase-mediated resistance: a biochemical, epidemiological and genetic overview. Curr. Pharm. Des. 19, 164–208. 10.2174/138161213804070320 22894615

[B4] Papp-Wallace K. M. Bonomo R. A. (2016). New beta-Lactamase Inhibitors in the Clinic. Infect. Dis. Clin. North. Am. 30, 441–464. 10.1016/j.idc.2016.02.007 27208767 PMC4980828

[B5] Sader H. S. Farrell D. J. Castanheira M. Flamm R. K. Jones R. N. (2014). Antimicrobial activity of ceftolozane/tazobactam tested against Pseudomonas aeruginosa and Enterobacteriaceae with various resistance patterns isolated in European hospitals (2011-12). J. Antimicrob. Chemother. 69, 2713–2722. 10.1093/jac/dku184 24917579

[B6] Tooke C. L. Hinchliffe P. Bragginton E. C. Colenso C. K. Hirvonen V. H. A. Takebayashi Y.. (2019). beta-Lactamases and beta-Lactamase Inhibitors in the 21st Century. J. Mol. Biol. 431, 3472–3500. 10.1016/j.jmb.2019.04.002 30959050 PMC6723624

